# Brain responses reveal young infants’ sensitivity to when a social partner follows their gaze

**DOI:** 10.1016/j.dcn.2013.09.004

**Published:** 2013-10-03

**Authors:** Tobias Grossmann, Sarah Lloyd-Fox, Mark H. Johnson

**Affiliations:** a Early Social Development Group, Max Planck Institute for Human Cognitive and Brain Sciences, Leipzig, Germany; b Centre for Brain and Cognitive Development, Birkbeck, University of London, UK

**Keywords:** Gaze, Joint attention, Infancy, Prefrontal cortex, fNIRS

## Abstract

Infants’ ability to follow another person's eye gaze has been studied extensively and is considered to be an important and early emerging social cognitive skill. However, it is not known whether young infants detect when a social partner follows their gaze to an object. This sensitivity might help infants in soliciting information from others and serve as an important basis for social learning. In this study, we used functional near-infrared spectroscopy (fNIRS) to measure 5-month-old infants’ frontal and temporal cortex responses during social interactions in which a social partner (virtual agent) either followed the infants’ gaze to an object (congruent condition) or looked to an object that the infant had not looked at before (incongruent condition). The fNIRS data revealed that a region in the left prefrontal cortex showed an increased response when compared to baseline during the congruent condition but not during the incongruent condition, suggesting that infants are sensitive to when someone follows their gaze. The findings and their implications for the development of early social cognition are discussed in relation to what is known about the brain processes engaged by adults during these kinds of social interactions.

## Highlights

• Brain responses measured during social gaze interactions in 5-month-olds.
• Infants’ brains detect when a social partner follows their gaze.
• Left prefrontal cortex involved in gaze following detection.
• This brain sensitivity is critical for social learning.

## 1 Introduction

Attending and responding to eye gaze is crucial for human social interactions. Specifically, eye gaze plays an important role in directing and coordinating attention during triadic interactions between self, other, and the environment. During a typical triadic interaction, a person may establish eye contact with another person and then direct that person's gaze to an object or event. The psychological process by which two people share attention toward the same object or event is referred to as joint attention. The ability to engage in triadic social interactions is thought to be critical for a wide range of human activities, supporting teaching, co-operation, and language learning (Csibra and Gergely, 2009, Tomasello, 1995, Tomasello et al., 2005). Moreover, impairments in joint attention are one of the earliest warning signs of neurodevelopmental disorders such as autism spectrum disorder (Charman, 2003). At the neural level, it has been shown that joint attention relies on the recruitment of the medial prefrontal cortex in adults (Schilbach et al., 2010, Williams et al., 2005), a brain structure that has been more generally implicated in social interaction, social cognition and theory of mind (Amodio and Frith, 2006, Schilbach et al., 2013).

In developmental behavioral work it has been shown that the ability to engage in joint attention emerges during the first year of life well before spoken language (Striano and Reid, 2006, Tomasello et al., 2005). In agreement with findings implicating medial prefrontal cortex in joint attention and theory of mind (Schilbach et al., 2013), behavioral differences in early joint attention abilities observed during infancy predict later differences in the more explicit understanding of others’ mental states assessed during childhood (Charman et al., 2001). Even though much progress has been made in understanding the behavioral emergence of joint attention during infancy (Carpenter et al., 1998, Striano and Stahl, 2005), very little is known about the brain substrate that supports joint attention in the developing infant. In a recent study, Grossmann and Johnson (2010) examined brain responses in 5-month-old infants’ prefrontal cortex during triadic social interactions using near-infrared spectroscopy (NIRS) (see Lloyd-Fox et al., 2010, for a description of the method and its use with infants). In order to investigate whether young infants engage specialized prefrontal brain processes when engaged in joint attention, in this study infants were presented with scenarios in which a social partner (virtual agent presented on a screen) (a) engaged in joint attention by gaze cueing the infants attention to an object after establishing eye contact [joint attention condition], (b) shifted gaze to an empty location [no referent condition], or (c) looked at an object without prior eye contact with the infant [no eye contact condition]. Only in response to the joint attention condition infants recruited a specific brain region within the prefrontal cortex, showing 5-month-old infants are sensitive to triadic interactions. Moreover, like adults, 5-month-olds recruited a prefrontal region localized in left dorsal prefrontal cortex when engaged in joint attention with another person (Schilbach et al., 2010), suggesting that young infants’ brains are tuned to share attention with others.

While Grossmann and Johnson's study (2010) provided first insights into the brain regions implicated in joint attention in infancy, an important outstanding question is whether infants are sensitive to when a social partner follows their gaze rather than how infants respond to joint attention initiated by an adult. This is a particularly critical question because (a) addressing this question can inform theories that posit that processes are shared and flexibly engaged by self and other initiated actions and interactions (Meltzoff, 2007, Schilbach et al., 2013), and (b) it may also speak to accounts postulating differences between responding to joint attention and the initiation of joint attention (Mundy and Newell, 2007). Specifically, a distinction has been made between: (a) responding to joint attention, that is, social gaze interactions that consist of infants’ responding to gaze cues of a social partner (following gaze) and (b) initiating joint attention, that is, interactions in which the infant initiates the social partner to follow gaze (Mundy and Newell, 2007). Recently, Schilbach and colleagues (2010) showed that in adults there are key brain regions, such as the left medial dorsal prefrontal cortex, involved in both responding to joint attention and to initiating joint attention. This suggests that adults flexibly engage specific brain processes that are shared between self and other initiated gaze interactions. Note that in Schilbach et al.’s study (2010) it was also shown that adults engage the ventral striatum only when initiating joint attention, indicating that activation of this brain region might be specific to self-initiated gaze interactions; however, brain activation from structures located as deep as the striatum cannot be examined with the neuroimaging method used in the current study (for more information see Section 4).

We examined 5-month-olds’ sensitivity to when a social partner follows their gaze. In addition to measuring brain responses from prefrontal cortex as in prior work (Grossmann and Johnson, 2010), we also assessed brain activity in temporal cortex including regions that have been shown to be involved in biological motion and eye gaze processing in infants and adults (Grossmann et al., 2008, Lloyd-Fox et al., 2009, Pelphrey and Morris, 2006). We measured infant brain responses using fNIRS during scenarios in which infants’ attention was first cued toward an object and then a social partner either followed the infants gaze to that object (congruent condition) or shifted attention to look at a different object (incongruent condition). Our prediction was that if young infants are sensitive to when a social partner follows their gaze then we will see greater brain activation during the congruent condition than during the incongruent condition in brain regions implicated in joint attention. More specifically, we hypothesized that if infants can flexibly engage the brain processes involved in joint attention regardless of whether the social gaze-based joint attention is driven by self or other (Meltzoff, 2007, Schilbach et al., 2013), they will show selective brain activation (left prefrontal) in the current study that is similar to what has been shown in prior work where infants followed someone's gaze (Grossmann and Johnson, 2010). Moreover, we hypothesized that during the incongruent condition infants will show brain activation in brain regions that are involved in working memory associated with the detection of a novel object.

## 2 Methods

### 2.1 Participants

The final sample consisted of 12 5-month-old infants (5 girls) aged between 137 and 158 days (*M* = 149.2 days). An additional five 5-month-olds were tested but not included in the final sample because they had too many motion artifacts resulting in too few usable trials for analysis (minimum number of 5 trials per condition). Please note that an attrition rate at this level is within the normal range for an infant fNIRS study (Lloyd-Fox et al., 2010). All infants were born full-term (37–42 weeks gestation) and with normal birth weight (>2500 g). All parents gave informed consent before the study.

### 2.2 Stimuli and procedure

Animated photo-realistic face stimuli were generated using Poser 6.0 (Curious Lab Inc.). This experiment consisted of two experimental conditions. In both conditions, the infant watched a person's face in the middle of the screen. Two objects (cars) were located to either side of the face. In order to attract infant's visual attention, after one second one of the two objects moved slightly and was highlighted by a red frame for 500 ms. This attention getting sequence was repeated once. Then, in the congruent condition the person on the screen raised her eyebrows and smiled while looking at the infant and then shifted the eyes toward the object the infant had looked at, and then looked back at the infant, and finally, turned the head toward the object. In the incongruent condition, the face did exactly the same as in the congruent condition, except that in this condition the face looked and turned toward the side of the object that the infant had not looked at. Eight different clips were created, face (female/male), object (left/right), and experimental conditions (congruent/incongruent). Infants sat on their parent's lap while watching the stimuli on a computer monitor within an acoustically shielded, dimly lit room. The visual angle of the faces presented subtended 38° × 25°, and each eye subtended 3° × 5°. An experimental trial was 7 s in duration. Trials from the two different conditions were pseudo-randomly distributed over the session with no more than two trials of the same condition occurring consecutively. The inter-trial interval was 12 s. Non-social moving visual stimuli were presented during the inter-trial interval to keep infants’ attention. Infants’ looking behavior was monitored by camera and then coded off-line. Only those data were included in the analysis were infants looked at the highlighted (attention-grabbing) object (see description above). Furthermore, infants had to look at the screen for at least 80% of the duration of the trial for the data of that trial to be included. Infants watched on average 22.3 trials (*SD* = 4.2).

### 2.3 Data acquisition and analysis

**Fig. 1:**
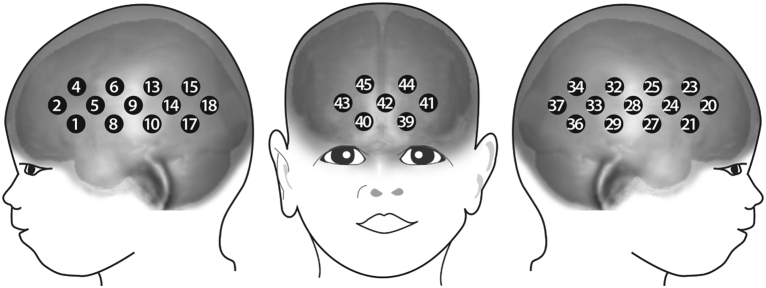
This figure illustrates the channel layout used in this fNIRS study with 5-month-old infants.

NIRS measurements were made using the UCL topography system (Everdell et al., 2005). The multi-channel system uses two wavelengths at 770 nm and 850 nm (sampling frequency 10 Hz). In custom-built arrays and head gear, ten optodes in a thirteen-channel (source-detector pairs) arrangement with an inter-optode separation of 20 mm were placed over the inferior frontal and temporal cortices on each hemisphere, and six optodes in a seven-channel (source-detector pairs) arrangement with inter-optode separation of 20 mm (channels 41, 42 and 43) and 25 mm (channels 39, 40, 44 and 45) were placed over the prefrontal cortex (see Fig. 1 for channel layout). Filtering and artifact rejection was performed according to an established procedure (for a detailed description of the analysis procedure, see Lloyd-Fox et al., 2009, Lloyd-Fox et al., 2011). Note that in the current study we used a different NIRS system with a different channel layout than in our prior work on joint attention in infants (see Grossmann and Johnson, 2010). Nonetheless, with respect to the comparability across studies, channel 44 in the current layout measured from a left prefrontal brain region that roughly matches the brain region specifically activated during joint attention in the prior study (Grossmann and Johnson, 2010). Cortical responses were assessed statistically by comparing average concentration changes (oxyHb and deoxyHb in μmol) within trials (5–15 s after stimulus onset) during the experimental conditions against baseline (activity during the inter-trial interval when non-social moving visual stimuli were presented) using two-tailed one-sample *t*-tests and between experimental conditions using two-tailed paired *t*-tests.

## 3 Results

**Fig. 2:**
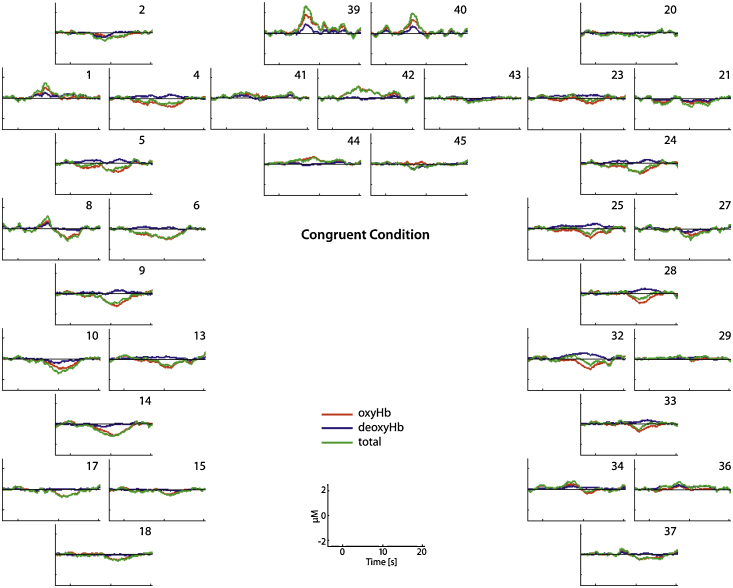
This figure shows the hemodynamic response function (HRF) during the congruent condition.

Our analysis of 5-month-old infants’ brain responses revealed that a region in left prefrontal cortex was sensitive to when a social partner followed the infant's gaze. As shown in Fig. 2, this brain region (channel 44) showed a significant increase in oxyHb when the congruent condition was compared to baseline (*t*[1,11] = 4.573, *p* = 0.0008), whereas the response to the incongruent condition was not significantly different from baseline (*t*[1,11] = 1.346, *p* = 0.205). The direct comparison between the brain responses to the congruent condition (*M* = 0.39; *SD* = 0.29) and the incongruent condition (*M* = 0.21; *SD* = 0.55) for this left prefrontal cortex region (channel 44) failed to reach significance (*t*[1,11] = 1.101, *p* = 0.294). Moreover, infants showed a greater decrease in deoxyHb in the congruent condition (*M* = −0.29; *SD* = 0.35) than in the incongruent condition (*M* = −0.03; *SD* = 0.53) in a right prefrontal region (channel 45; *t*[1,11] = 2.293, *p =* 0.042). A greater decrease in deoxyHb is thought to indicate cortical activation (Obrig and Villringer, 2003). There was also more widespread decreases in oxyHb relative to baseline during the congruent condition than during the incongruent condition in a number of channels in the left and right hemisphere (channels deactivated during congruent condition 2, 4, 5, 13, 17, 24, 28; channels deactivated during incongruent condition 14, 17, 33; all channels *p* < 0.05).

**Fig. 3:**
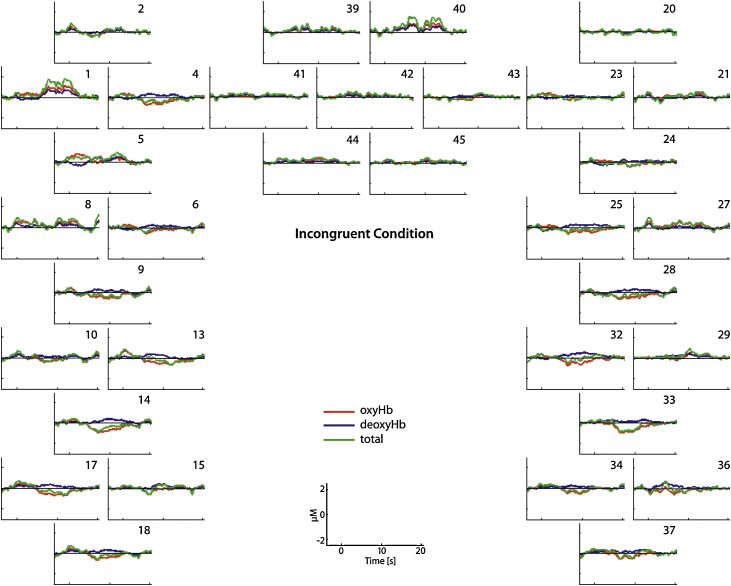
This figure shows the hemodynamic response function (HRF) during the incongruent condition.

Our analysis further revealed that a brain region within the left inferior frontal cortex was sensitive to when the social partner looked at an object that the infant had not previously looked at. As shown in Fig. 3, this brain region (channel 1) showed a significant increase in oxyHb when the incongruent condition (*M* = 0.75; *SD* = 1.13) was compared to baseline (*t*[1,11] = 2.447, *p* = 0.032), whereas the response to the congruent condition (*M* = 0.52; *SD* = 1.12) was not significantly different from baseline (*t*[1,11] = 1.627, *p =* 0.139). Furthermore, there was a region within the right posterior temporal cortex (channel 33) that showed a significantly greater decrease in oxyHb relative to baseline during the incongruent condition (*M* = −0.67; *SD* = 0.44) when compared to the congruent condition (*M* = −0.09; *SD* = 0.78), *t*[1,11] = 2.671, *p =* 0.021.

## 4 Discussion

Our results revealed that a region in the left prefrontal cortex showed an increased response when compared to baseline during the congruent condition but not during the incongruent condition, suggesting that 5-month-old infants are sensitive to when someone follows their gaze. This sensitivity might help infants in soliciting information from others and hence may serve as an important foundation for social learning. From a developmental perspective, this finding is in line with theories emphasizing the importance of the early emergence of social cognitive abilities required to engage in triadic social interactions (Csibra and Gergely, 2009, Tomasello et al., 2005) and supports theories positing a link between processes implicated in actions performed by self and by others (Meltzoff, 2007). From a neuroscience perspective, this finding further strengthens accounts that – in contrast to the commonly held notion of a late maturation of prefrontal cortex functions – assign a pivotal functional role to the prefrontal cortex in infant cognition in general (Grossmann, 2013a) and medial prefrontal cortex in infant social cognition in particular (Grossmann, 2013b). The current findings thus provide important insights into the neurodevelopmental basis of social cognitive functioning.

Our findings show that the infant prefrontal brain response during the congruent condition was observed in the left hemisphere, which is not only in line with the adult functional magnetic resonance imaging (fMRI) work (Schilbach et al., 2010) but also corresponds with findings showing that greater cortical activation in the left prefrontal cortex is positively correlated with children's tendency to initiate joint attention (Caplan et al., 1993, Mundy et al., 2000). With respect to the lateralization of the prefrontal brain response, it is noteworthy that prior work with infants and adults has shown that left prefrontal cortical activation is indicative of a motivation to approach (Davidson and Fox, 1982, Fox, 1991, Harmon-Jones, 2003). This raises the possibility that during the congruent condition infants responded with the motivation to approach the social partner that followed their gaze, whereas this motivation was absent when the social partner did not follow gaze. Such an effect on motivational systems may serve a vital function in guiding infant social behavior by driving infants to interact with, learn from and share experiences with cooperative partners (Tomasello et al., 2012).

The finding that left prefrontal cortex plays a role in detecting when a social partner follows gaze is based on the results obtained when comparing the congruent condition to baseline, while a direct comparison between the congruent and incongruent condition failed to reach significance. This might be explained by the fact that in both conditions the interaction is triadic in nature (characterized by eye contact with the infant and a referential look to an object) and this left prefrontal brain region has been shown to be sensitive to triadic interactions in previous work with infants of the same age (Grossmann and Johnson, 2010), thus resulting in an activation of this brain region in both conditions. However, the fact that we find a significant difference when looking at the contrast with baseline in the congruent condition but not in the incongruent condition suggests that there is something over and above the triadic nature of the interaction that engages this region. Clearly, more work is needed to further explicate the exact functional role that this region plays during social gaze-based interactions in infancy.

Another important issue is how the current findings with infants relate to prior work with adults. In particular, Schilbach and colleagues (2010) assessed the neural basis of the distinction between responding to joint attention and initiating joint attention in adults, and found that while there are regions commonly involved in both kinds of joint attention, such as the left medial dorsal prefontal cortex, the ventral striatum appears to be specifically engaged only when an adult initiates joint attention. This specific involvement of the ventral striatum has been argued to be the basis of the rewarding affective experience associated with directing someone else's gaze. Even though the work with infants found that there is a shared brain basis for other initiated (Grossmann and Johnson, 2010) and self initiated (current study) joint attention as shown in the left prefrontal brain responses, fNIRS as used in the infant work is not suitable for detecting responses from brain structures located as deep in the brain as the ventral striatum (Lloyd-Fox et al., 2010). With respect to the distinction between responding and initiating joint attention, it will be important to improve the current design in two ways. First, in line with previous work with older children (Mundy and Newell, 2007), future studies should include a condition examining infant brain activation when infants voluntarily (not externally cued as in the current study) shift gaze to an object before the social partner looks at the same object in a purely self-initiated joint attention fashion (Schilbach et al., 2013). Second, other neurophysiological or behavioral measures indicative of the affective response of the infant should be included in order to investigate whether the nature of the social gaze interaction is associated with differences in positive affect.

We additionally observed a significant effect in a right prefrontal region in which the decrease in deoxyHb was greater in response to the congruent condition than to the incongruent condition. A decrease in deoxyHb is taken as evidence for activation of a brain region (Obrig and Villringer, 2003), with some methods such as fMRI fundamentally relying on a decrease in deoxyHb as a measure of activation. This suggests that the right prefrontal cortex might be more involved during the congruent condition when infants’ gaze was followed. However, in order to firmly conclude that a certain brain region is activated one would expect an increase in oxyHb to be accompanied by a concomitant decrease in deoxyHb. Because this was not the case here, this finding is difficult to interpret. In general, there have been only very few fNIRS studies with infants that report reliable deoxyHb results, and the nature of the hemodynamic response during infancy and potential differences between infants and adults in this regard are still being debated (see Lloyd-Fox et al., 2010).

In addition to the main findings, we also observed widespread deactivation in bilateral inferior frontal and temporal regions during the congruent condition and in bilateral temporal regions during the incongruent condition (as indexed by a decrease in oxyHb when compared to baseline). It is difficult to interpret deactivation effects because (a) deactivation depends on what is presented during baseline, and (b) it is not entirely clear what a decrease in oxyHb in a particular region indicates and what functional relevance it may have (Lloyd-Fox et al., 2010). One possibility is that the detection of a social partner following one's own gaze requires increased social cognitive processing resources in the prefrontal cortex, and this in turn directly or indirectly suppresses other processes in more posterior regions of the brain leading to a deactivation (Schilbach et al., 2008). Such an inverse relationship between prefrontal and posterior brain regions has been reported in a variety of learning studies with adults (Gilbert and Sigman, 2007, Sigman et al., 2005), suggesting that this might be a more general neurocomputational principle. However, further work is required to clarify this issue.

Finally, our results also revealed that a region in the left inferior frontal cortex showed an increased response when compared to baseline during the incongruent condition but not during the congruent condition, suggesting that this region is involved in infants’ detection of the person's gaze being directed at an object different or novel from what they have looked at before. The left inferior frontal cortex has been shown to be involved in object working memory processes in adults (Nee et al., 2013), suggesting that infants’ detection of a novel object through a social interaction relies on working memory processes in the inferior frontal cortex. In addition, there was a region in the right posterior temporal cortex that in prior work has been shown to be sensitive to biological motion and eye gaze direction (Grossmann et al., 2008, Lloyd-Fox et al., 2009) that showed an increased deactivation during the incongruent condition than during the congruent condition. As argued above, such deactivations are hard to interpret. However, one possibility, as shown in prior work with adults (Pelphrey et al., 2003), is that incongruent looks of a social partner at an object evoke brain responses that are critically different from congruent looks in eye gaze sensitive cortical regions in the right posterior temporal cortex, suggesting that not only the movement of the eyes but also the context in which it occurs is relevant for this brain region in infant temporal cortex.

Taken together, the current findings provided critical insights into the social cognitive capacities that infants possess with regard to making sense of social interactions during which they have to keep track of a social partners eye gaze behavior. In particular, we have shown that a brain region within the left prefrontal cortex is sensitive to when a social partner follows the infant's gaze, whereas a region with the left inferior frontal cortex is sensitive to when a social partner acts incongruently to the infant. The finding that such young infants can distinguish between these social interactions and selectively recruit specific brain regions suggest that this ability is of pivotal significance for early social cognitive development and learning.

## Conflict of interest statement

The authors declare no conflict of interest.
